# Pre- and Post-partum Berberine Supplementation in Dairy Goats as a Novel Strategy to Mitigate Oxidative Stress and Inflammation

**DOI:** 10.3389/fvets.2021.743455

**Published:** 2021-10-14

**Authors:** Navid Ghavipanje, Mohammad Hasan Fathi Nasri, Seyyed Homayoun Farhangfar, Seyyed Ehsan Ghiasi, Einar Vargas-Bello-Pérez

**Affiliations:** ^1^Department of Animal Science, Faculty of Agriculture, University of Birjand, Birjand, Iran; ^2^Department of Veterinary and Animal Sciences, Faculty of Health and Medical Sciences, University of Copenhagen, Frederiksberg, Denmark

**Keywords:** antioxidant, berberine, inflammation, transition goats, oxidative stress

## Abstract

As in dairy cattle, goats during the transition period face risk factors, in particular negative energy balance (NEB), inflammation, and impairment of the antioxidant response. The current study determined the effects of pre- and post-partum berberine (BBR) supplementation on antioxidant status and inflammation response during the transition period in dairy goats. Twenty-four primiparous Saanen goats were randomly divided into four groups: control (CON, without BBR) and supplemented with 1 g/day BBR (BBR1), 2 g/day BBR (BBR2), or 4 g/day BBR (BBR4). The blood samples were collected weekly from 21 days pre-partum to 21 days post-partum. Compared with CON, supplementation with either BBR2 or BBR4 decreased (*P* ≤ 0.05) the levels of plasma non-esterified fatty acids (NEFA) at kidding and thereafter an increased (*P* ≤ 0.05) the plasma levels of glucose and insulin. Following BBR ingestion, blood antioxidant status elevated throughout the transition period, so that total antioxidant capacity (TAC), glutathione peroxidase (GSH-Px), superoxide dismutase (SOD), and catalase activity were increased (*P* ≤ 0.05) and plasma malondialdehyde (MDA) was decreased (*P* ≤ 0.05). Likewise, paraoxonase (PON) was reduced (*P* ≤ 0.05) in goats fed BBR2 and BBR4. The levels of haptoglobin, ceruloplasmin, and bilirubin were reduced (*P* ≤ 0.05) by BBR2 and BBR4 immediately before kidding and thereafter. The results demonstrated that supplementation of either 2 or 4 g/day BBR enhanced antioxidant capacity and immune function of transition goats and improved post-partum performance showing its beneficial effect to mitigate oxidative stress and inflammation during the transition period in dairy goats.

## Introduction

The periparturient period, also known as the transition period, usually comprises 3 weeks pre-partum to 3 weeks post-partum and is accompanied by severe metabolic, hormonal, and immunological changes in preparation for parturition and the production of colostrum and milk (1, 2). It is well established that the adequate preparations are essential for goats during the transition period to coordinate the challenges of delivery of kid, the onset of lactation, and metabolic dysfunction (3, 4). Furthermore, the energy intake lags behind the increasing nutrient demands and leads to a negative energy balance (NEB). Dysregulated immune responses and oxidant/antioxidant imbalance have been proposed as a consequence of NEB that ultimately affects the productive and reproductive performance of dairy goats (5, 6), similar to cows (7). Normally, in the blood from dairy goats during the transition period as well as in dairy cows, the increase in lipid peroxidation leads to a decrease in plasma antioxidant concentrations and a decrease in antioxidative enzyme activity (1, 6, 8).

Moreover, the parturition process commonly entails immunosuppression which increases the expression and release of inflammatory mediators (7) that may stimulate lipolysis, increasing the releasing of the non-esterified fatty acids (NEFA), and promoting oxidative stress (2). Immunosuppression may reduce feed intake and increase energy losses (2, 9). In fact, recent evidence shows that an over response occurs in both inflammatory [e.g., a decrease of negative acute phase proteins (neg APPs) such as albumin, cholesterol, paraoxonase, retinol-binding protein] and metabolic [e.g., a decrease in plasma glucose and an increase in NEFA, b-hydroxybutyrate (BHBA), and reactive oxygen metabolites] indices in transition cows (2, 6, 9)]; however, limited information is available for dairy goats during the transition period.

Plant secondary metabolites have been suggested as immunological and antioxidant modulators that might exert favorable effects on oxidative status and overall health of animals (10, 11). Berberine (BBR) is a yellow alkaloid present in various medicinal plants, most notably *Coptis, Hydrastis*, and *Berberis* (12). BBR is a quaternary benzylisoquinoline alkaloid, a relevant molecule in pharmacology and medicinal chemistry. In fact, it is known for the synthesis of several bioactive derivatives by means of condensation, modification, and substitution of functional groups in strategic positions for the design of new, selective, and powerful clinical drugs (13).

BBR has been studied for more than eight decades as the first review on its isolation and chemistry was published in 1926 (14). BBR has several beneficial biological properties including antioxidant, antimicrobial, anticancer, anti-inflammatory, and glucose lowering (13, 15). Numerous reports in both cell culture (16) and animal models (17) have also highlighted the inhibitory effect of BBR on oxidative stress. The antioxidant activity of BBR is well documented as it altered the levels of antioxidant enzymes and oxidative stress markers such as glutathione (GSH), a lipid oxidation product that is reduced in oxidative stress, and malondialdehyde (MDA) that is increased in oxidative stress (18–20).

The anti-inflammatory activity of BBR was observed both *in vitro* and *in vivo* using animal models and was noted by the reduction of pro-inflammatory cytokines as well as acute phase proteins (15, 20, 21). Recent studies showed that BBR decreased pro-inflammatory cytokines such as TNF-α, IL-13, IL-6, IL-8, and IFN-γ through multiple cellular kinases as well as signaling pathways such as AMPK, MAPKs, Nrf2/HO, and NF-κB that were verified to be pivotal for BBR in reducing oxidative stress and inflammation (15, 22). Also, Li et al. (23) reported that the cellular pathways of BBR in inhibiting inflammation are apparently shared in part with its antioxidant pathways. Information on the use of BBR in ruminants is scarce, but several studies have been conducted using non-ruminant models indicating that BBR promisingly exhibits antioxidant and anti-inflammatory features (15, 17). In this way, Zhang et al. (14) showed the beneficial effects of supplementing 100 mg/kg of BBR on immunity and antioxidant activities in broilers. In addition, an improved glucose and insulin metabolism in rats has been reported following BBR (50 and 100 mg/kg) supplementation (24). de Oliveira et al. (25) reported that 50 or 100 mg/kg body weight (BW) BBR reduces oxidative stress by prevention of ROS production. Likewise, inflammation markers of diabetic mice were ameliorated by supplementation with 50 mg/kg BW BBR (26).

As previously mentioned, in dairy goats, the transition from late pregnancy to early lactation not only poses metabolic challenges, because of the NEB, inflammation, and oxidative stress, but also gives high prolificacy and improved milk production in modern dairy goats, and the risk of developing the aforementioned metabolic complications were exacerbated, both pre- and post-partum. In this way, appropriate strategies to alleviate NEB, inflammation, and oxidative stress during the transition period are a top priority in dairy goat nutritional management. This study hypothesized that BBR supplementation to dairy goats during the transition period could mitigate oxidative stress and inflammation. As such, supplemental BBR will have a positive effect on animal health and oxido-inflammatory status. Hence, supplementation of berberine to dairy goats in the current study is an attempt to mitigate oxidative stress and inflammation markers reflected by circulating blood metabolites and enzymes and to decrease the incidence of periparturition metabolic challenges.

## Materials and Methods

### Animals, Diets, and Treatments

This study followed the guidelines of the Iranian Council of Animal Care (27) (ID 19293). The study was carried out at the experimental farm of the Faculty of Agricultural Research, University of Birjand, Iran. Twenty-four first lactating Saanen goats selected from the experimental herd according to BW (45 ± 3.5 kg, mean ± SD) and body condition score (BCS) (3 ± 0.5, mean ± SD) were enrolled at 50 days before their expected kidding date and stayed in the trial until 21 days after kidding. The first 29 days were used for adaptation to avoid possible alterations because of diet changes, and the remaining 42 days were used for measurements. During the experiment, all goats were housed in individual pens (1.8 m × 1.6 m) in a ventilated enclosed barn. Diets were formulated to be isocaloric and isonitrogenous and to meet NRC (28) nutritional requirements of each period (pre- and post-partum). The diet provided during the pregnancy period contained 18.5% crude protein (CP) and 2.60 Mcal ME (dry matter basis), and the diet provided during the lactation period contained 15.5% CP and 2.90 ME per kg dry matter. Goats were fed *ad libitum* total mixed rations (consisted of 45% forage and 55% concentrate, DM basis) that were mixed daily and provided twice per day in equal amounts at 06:00 and 16:00 h (Table 1).

**Table 1:** Ingredients and nutrient composition (DM basis) of pre- and post-partum diets.

	**Diets**	
	**Pre-partum^a^**	**Post-partum^b^**
**Ingredient (% of DM)**		
Alfalfa hay	4.00	29.5
Corn silage	34.3	10.8
Wheat straw	17.9	5.00
Barley grain, ground	7.70	10.8
Corn grain, ground	31.5	22.2
Soybean meal	1.00	17.0
Wheat bran	1.80	2.20
Calcium carbonate	0.90	1.00
Minerals and vitamins premix^c^	0.90	0.50
Salt	0.00	1.00
**Chemical composition**		
ME, Mcal/kg of DM^d^	2.60	2.90
Crude protein (% DM)	18.5	15.5
Ether extract (% DM)	2.50	2.50
Ash (% DM)	7.60	8.00
Neutral detergent fiber (% DM)	43.0	37.3
Non-fibrous carbohydrates (% DM)^e^	38.0	36.7

a *From 50 days before parturition until kidding*.

b *From kidding until 21 days of lactation*.

c *Containing vitamin A (250,000 IU/kg), vitamin D (50,000 IU/kg), vitamin E (1,500 IU/kg), manganese (2.25 g/kg), calcium (120 g/kg), zinc (7.7 g/kg), phosphorus (20 g/kg), magnesium (20.5 g/kg), sodium (186 g/kg), iron (1.25 g/kg), sulfur (3 g/kg), copper (1.25 g/kg), cobalt (14 mg/kg), iodine (56 mg/kg), and selenium (10 mg/kg)*.

d *Cornell Net Carbohydrate and Protein System predicted based upon measured feedstuff nutrient composition and actual DMI using SRNS (29)*.

e *Non-fibrous carbohydrates (NFC) were estimated according to the equation: NFC = 100 – (NDF + CP + EE + Ash) (28)*.

Although there are no comparable data regarding the effects of BBR in ruminant studies, previous reports (15, 19, 25, 30) indicated that doses from 25 to 100 mg/kg BW dietary BBR can positively impact non-ruminant species and that the dosage level can affect the levels of responses (31, 32). Hence, in the current study, animals were randomly assigned to one of four dietary treatments arranged in a completely randomized design with the following treatments: a control (CON), receiving pre-partum and post-partum basal diet without BBR; and three groups were fed pre-partum and post-partum basal diets supplemented with BBR at the levels of 1, 2, and 4 g/day. Dietary treatments correspond to 0, 25, 50, and 100 mg/kg BW dietary BBR, respectively. BBR was purchased from Bulk Supplement Factory (Bulk Supplements, Eastgate, Henderson, USA), and based on the catalog, this product is Pure Berberine HCL powder and had no other ingredients. To ensure full treatment intake, BBR was encapsulated in gelatin capsules (Irancapsul, Tehran, Iran). The BBR capsules were orally administrated to each goat before morning feeding with a balling gun (Pars Khavar, Tehran, Iran), while the control group received empty ones.

### Sample Collection and Measurements

Manual weighing of feeds and refusals were performed daily from each goat. The TMR and refusal samples were taken weekly before the morning feeding and frozen at −20°C for later analysis. The blood samples (10 ml/goat) were collected before morning feeding, from the jugular vein using tubes containing Li-heparin as anticoagulant (RotexMedica, Germany) on days −21, −14, −7, 0, 7, 14, and 21 relative to the expected kidding. The blood samples were centrifuged at 3,000 × *g*, 15 min at room temperature, and stored in plastic microtubes at −80°C for later analysis.

### Laboratory Analysis

Samples of TMR and orts were separately pooled and ground in a hammer mill with a 1-mm screen (Arthur Hill Thomas Co., Philadelphia, PA). Dry matter of the samples was determined after oven drying for 72 h at 55°C and then analyzed (three replicates) according to the procedures of AOAC (33) for ash (967.05), CP (Kjeldahl N × 6.25, 990.03), and ether extract (EE, 945.16). Neutral detergent fiber (NDF) was measured (Fibertec 1010, Tecator, Sweden) as described by Van Soest et al. (34).

Plasma samples were analyzed for concentrations of albumin, total protein, creatinine, triacylglycerol, cholesterol, glucose, high-density lipoprotein (HDL), and blood urea-N, using commercial kits (Pars Azmun Co. Ltd., Tehran, Iran) by an autoanalyzer (BT 1500, Biotecnica SpA, Rome, Italy). Total antioxidant capacity (TAC) was measured using ABTS^+^ [2,2′-azino-di-(3-ethylbenzthiazoline sulfonate)] radical formation with the commercially available Randox kit (Randox Lab., Ltd., Crumlin, County Antrim, UK). The principle of the assay is the ability of aqueous and lipid antioxidants in the plasma specimens to inhibit the oxidation of ABTS^+^ that has a stable blue-green color, which is measured at 600 nm. The concentrations of BHBA and NEFA were measured calorimetrically by commercial kits (Randox Laboratories Ltd., Ardmore, UK) using the same autoanalyzer.

Plasma haptoglobin was detected using commercial test kits (Randox Laboratories Ltd., Ardmore, UK) that measure hemoglobin peroxidase activity, which is in direct proportion to the quantity of haptoglobin. Plasma paraoxonase (PON) activity was measured by adapting the method of Ferre et al. (35). Briefly, 8 μl of plasma added to 125 μl of ultrapure water and 125 μl of assay buffer were incubated at 37°C. The rate of hydrolysis of paraoxon to *p*-nitrophenol was measured by monitoring the increase in absorbance at 405 nm, using a molar extinction coefficient of 18,050 L/mol/cm. The unit of PON activity (U/ml) is defined as 1 nmol of *p*-nitrophenol formed per minute under the assay conditions.

The serum insulin level was measured using an enzyme-linked immunosorbent assay kit (Kit number: 2425-300B, Monobind Inc., CA, USA). Intra- and interassay coefficients of variation for measuring insulin were 6.9 and 8.2%, respectively. The level of MDA in plasma was determined using the thiobarbituric acid method (TBARS) according to Placer et al. (36). The enzymatic activities of glutathione peroxidase (GSH-Px), superoxide dismutase (SOD), and catalase (CAT) were measured using a Randox kit (Randox Laboratories Ltd., Ardmore, UK) according to the procedures of the manufacturers. The GSH-Px, SOD, and CAT activities were expressed as U/g of hemoglobin.

### Statistical Analysis

The effects of supplementation with BBR on all examined parameters were statistically analyzed using the MIXED procedure of Statistical Analysis System 9.2 software (SAS Institute Inc.) (37). The model for pre- and post-partum included the fixed effects of treatments (different levels of BBR supplementation), sampling day, and the random effect of goat nested within treatment and the residual error. Interactions between treatments and sampling times were included in the model, if they were significant. Three covariance structures were tested (autoregressive 1, spatial power, and unstructured), and the one resulting in the lowest Akaike information criterion was chosen. The REPEATED procedure was used for all variables and least square means (LSM) separation between time points was performed using the PDIFF statement by Tukey's test and presented as LSM ± SEM. A polynomial contrasts analysis was employed to determine the linear and quadratic effects of BBR dose. A threshold of significance was set at *P* ≤ 0.05; trends were declared when the *P*-value was > 0.05 and ≤ 0.10.

## Results

### Biomarkers of Energy Metabolism

**Figure 1:**
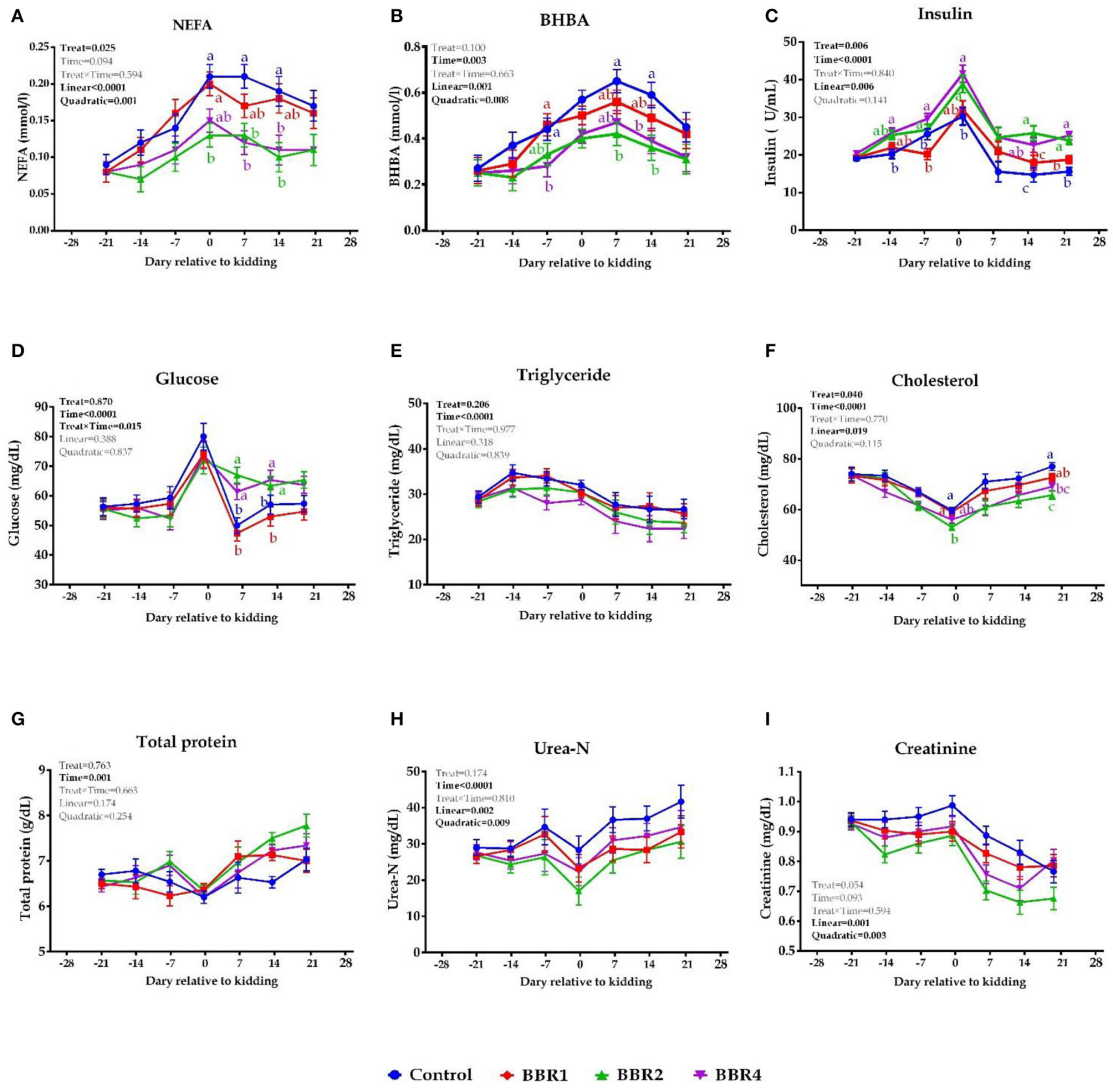
Means (± SEM) of **(A)** NEFA, **(B)** BHBA, **(C)** insulin, **(D)** glucose, **(E)** triglyceride, **(F)** cholesterol, **(G)** total protein, **(H)** urea-N, and **(I)** creatinine in Saanen dairy goats supplemented with different levels of BBR during the transition period. ^a−*c*^Values with different superscript letters at the same time point are significantly different (*P* ≤ 0.05).

Both BBR2- and BBR4-supplemented doses showed a significant lower plasma NEFA at days 0, 7, and 14 relative to the parturition (*P* ≤ 0.05, Figure 1A). Supplementation of BBR2 and BBR4 reduced the BHBA concentration on days −7, 7, and 14 (*P* ≤ 0.05) (Figure 1B), and also a tendency of decrease was observed in BBR2- and BBR4-supplemented goats at kidding (*P* = 0.10). Increased insulin levels were detected in response to BBR supplementation throughout the transition period with the exception of days −21 and 7 (*P* ≤ 0.05, Figure 1C). Goats receiving both BBR2 and BBR4 treatments had greater plasma glucose during the post-partum period (*P* ≤ 0.05, Figure 1D). At days 0 and 21, supplementation with either 2 or 4 g of BBR reduced the cholesterol concentration doses (*P* ≤ 0.05, Figure 1F). No significant changes in plasma concentrations of triglyceride (TG), total protein (TP), urea-N, and creatinine were detected in response to BBR supplementation during the pre- and post-partum (*P* > 0.05). Overall, BBR-supplemented goats showed lesser plasma concentration of NEFA, BHBA, and cholesterol as well as greater levels of insulin and glucose (main effect *P* ≤ 0.05, Figure 1). Except for creatinine, all values were significantly different throughout the transition period (time *P* ≤ 0.05), but no treatment by time interaction was observed (*P* > 0.05; Figure 1). Moreover, a quadratic effect of BBR on NEFA, BHBA, urea-N, and creatinine was observed (*P* ≤ 0.05).

### Biomarkers of Oxidative Stress and Antioxidant Status

**Figure 2:**
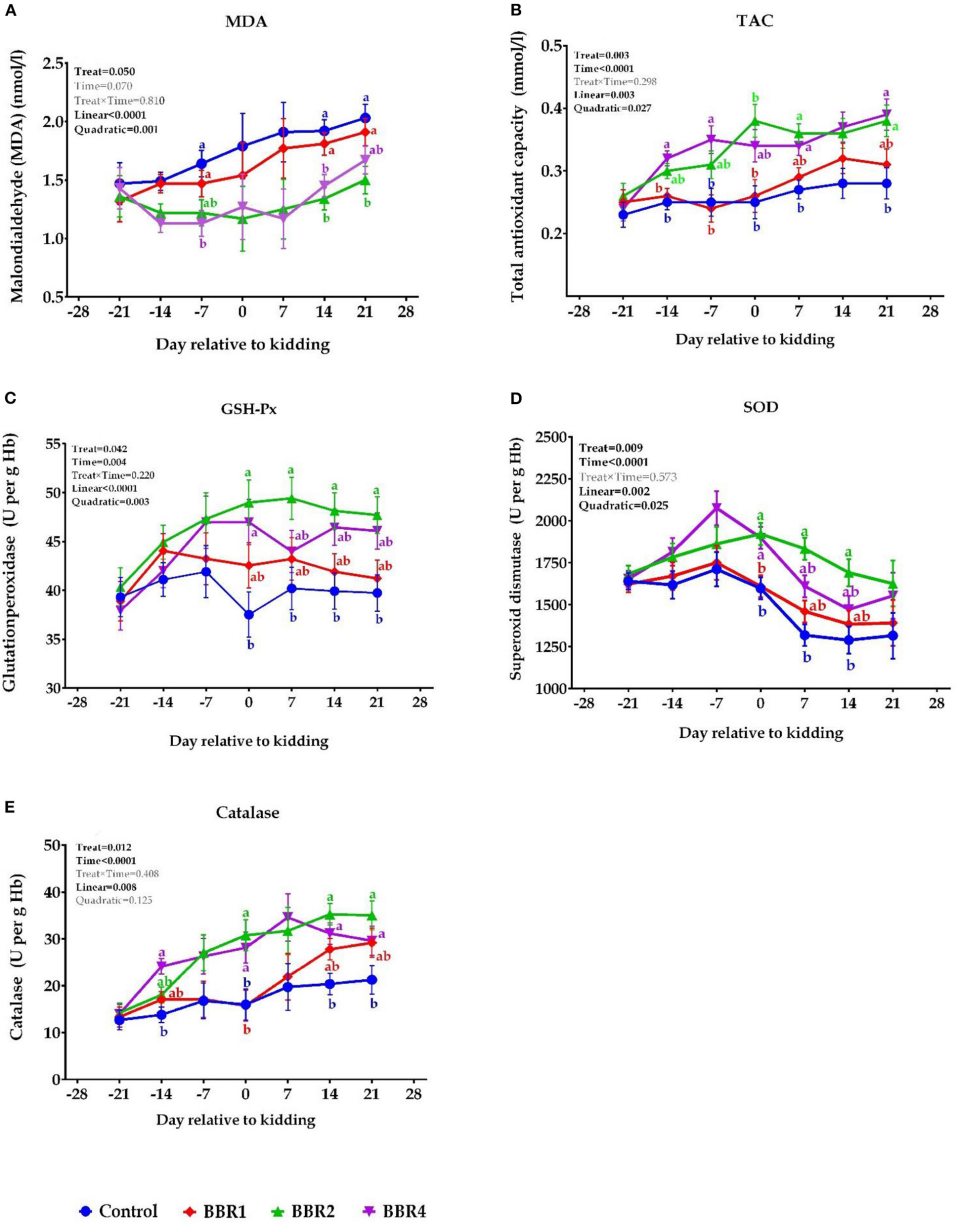
Dynamic effects of BBR supplementation on the levels of **(A)** MDA, **(B)** TAC, **(C)** GSH-Px, **(D)** SOD, and **(E)** catalase in Saanen dairy goats supplemented with different levels of BBR during the transition period. ^a, b^Values with different superscript letters at the same time point are significantly different (*P* ≤ 0.05).

The dynamic effects of BBR supplementation on biomarkers of oxidative stress over the 6-week transition period are shown in Figure 2. At days −21, −14, 0, and 7 relative to the parturition, non-significant differences were observed for MDA (*P* > 0.05), whereas BBR2- and BBR4-treated goats showed lower MDA concentration on days −7, 14, and 21 (*P* ≤ 0.05) (Figure 2A). Overall, supplementing BBR increased TAC levels throughout the transition period (Figure 2B), where the greatest plasma TAC concentration was found on days −14, −7, and 21 for BBR4 and on days 0, 7, and 14 for the BBR2 group (*P* ≤ 0.05).

As shown in Figure 1C, the pre-partum GSH-Px concentration was not affected by treatments; however, weekly GSH-Px concentration was significantly higher in BBR-supplemented goats at the kidding and thereafter, led by BBR2 (*P* ≤ 0.05). Regarding the effect of treatments, a similar pattern was observed for SOD and CAT, meaning that after parturition, their concentrations were increased in the BBR-fed goats (*P* ≤ 0.05). Indeed, an upward trend of changes was observed in CAT with the advent of parturition and following the occurrence of lactation. As a whole, BBR supplementation decreased plasma MDA concentrations and elevated TAC, GSH-Px, SOD, and CAT levels (main effect *P* ≤ 0.05, Figure 2). No interaction effect was detected between BBR and week (*P* > 0.05). Except for MDA, all enzymes were significantly different during the 6 weeks of the study (time *P* ≤ 0.05). In addition, there were quadratic effects of BBR doses on MDA, TAC, SOD, and CAT levels (*P* ≤ 0.05).

### Biomarkers of Inflammation and Liver Functionality

**Figure 3:**
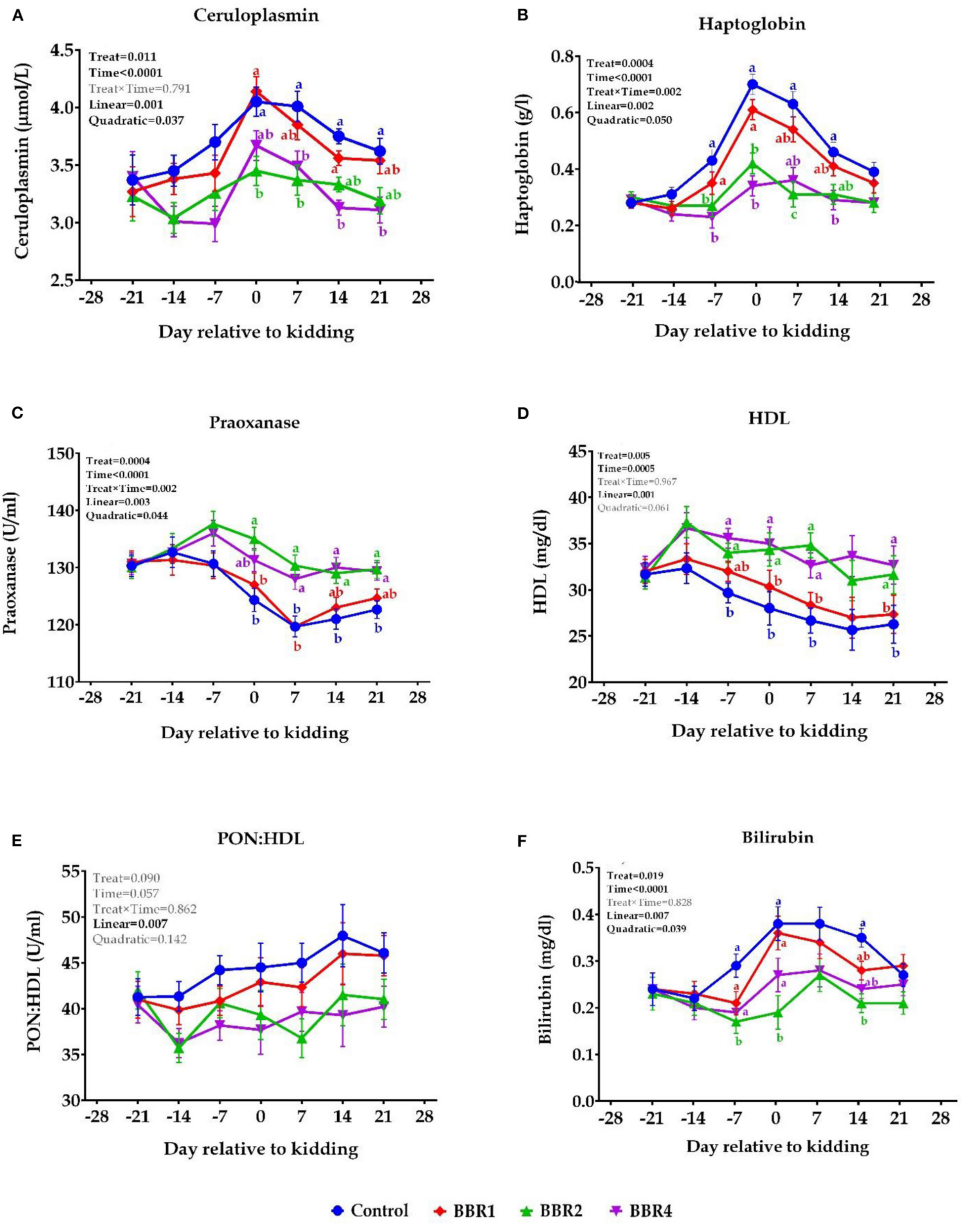
Means (± SEM) of biomarkers of positive acute-phase protein [**(A)** ceruloplasmin and **(B)** haptoglobin] and negative-acute phase protein [**(C)** paraoxonase, **(D)** HDL, **(E)** paraoxonase/HDL, and **(F)** bilirubin] in Saanen dairy goats supplemented with different levels of BBR during the transition period. ^a, b^Values with different superscript letters at the same time point are significantly different (*P* ≤ 0.05).

Ceruloplasmin and haptoglobin (Figures 3A,B, respectively) had the greatest values at parturition; thereafter, a gradual reduction was observed for all four groups. Over the pre-partum period, the ceruloplasmin concentration did not differ between groups (*P* > 0.05); however, supplementation with both BBR2 and BBR4 decreased ceruloplasmin concentration at the parturition and the post-partum period (*P* ≤ 0.05); the greatest decline in the ceruloplasmin was observed in the BBR2 group, followed by the BBR4 group on days 0 and 7 and vice versa in days 14 and 21 (*P* ≤ 0.05). During the pre-partum period, the plasma haptoglobin concentration kept rising and peaked at day 0 for all groups, which is more pronounced in CON, and then reduced progressively from 0 to 21 days. BBR supplementation (2 and 4 g/day) decreased haptoglobin concentration immediately before parturition (day −7), at kidding day, and thereafter until day 21 compared with the CON (*P* ≤ 0.05).

There were no significant differences for paraoxonase concentration between groups before the parturition (Figure 3C), but it showed a sharp decrease immediately after the parturition in all groups; however, using supplemental BBR2 and BBR4 revived its concentration at the kidding and thereafter (*P* ≤ 0.05), which was more pronounced for BBR2. Plasma concentrations of HDL decreased during the last few days of pregnancy, and this downward trend continued after parturition in all treatments, which was more severe for the CON group. Following BBR supplementation, no difference was observed in HDL during the first week of transition (day −21 until −14) (Figure 3D, *P* > 0.05); however, both BBR2- and BBR4-fed goats showed significantly higher HDL levels immediately before kidding (day −7) and thereafter (*P* ≤ 0.05), except for day 14. However, PON:HDL were not affected (Figure 3E, *P* > 0.05). Bilirubin concentration increased after the first week of lactation and the peak value appeared at parturition in all groups (Figure 3F). However, values for the BBR2 group remained lower than the CON in days −7 and 0 relative to the kidding (*P* ≤ 0.05). In addition, all BBR-fed groups had lower plasma bilirubin in day 14 after parturition compared with the CON (*P* ≤ 0.05).

Whether it was before or after parturition, supplementation with both BBR2 and BBR4 enhanced paraoxonase and HDL and reduced ceruloplasmin and haptoglobin concentrations (main effect *P* < 0.05). All values were significantly different in 6 weeks (time *P* < 0.001); moreover, haptoglobin and paraoxonase showed interaction with weeks (interaction *P* = 0.002). Except for HDL, all biomarkers of inflammation and liver functionality were quadratically affected by BBR (*P* ≤ 0.05).

## Discussion

To our knowledge, not enough data are available with regard to the transition period of dairy goats as well as possible approaches to mitigate oxido-inflammatory status. Relatively few studies have examined periparturient status in dairy goats which mainly focused on a few blood parameters with respect to parity (3, 5) and litter size (38), or temporal variations in circulating levels of selected hormones and metabolites (4, 6), together with some performance indicators. This article is part of a larger study that evaluated the effects of BBR supplementing on performance, glucose metabolism, insulin sensitivity, inflammation, and oxidative stress status in periparturient dairy goats. Our companion paper (39) on intake, lactation performance, and energy metabolism revealed that BBR supplementation increases DMI with enhancement of milk production and BSC and demonstrates that it could be a useful tool to ameliorate NEB and metabolic-related stress. In the current study, we assessed the effect of BBR supplementation during the transition period on blood biomarkers related to energy metabolism and inflammatory and oxidative stress indicators in pre- and post-partum dairy Saanen goats.

### Biomarkers of Energy Metabolism

During late gestation and early lactation, body fat mobilization is followed by NEB that is characterized by increases in circulating concentration of NEFA (1). At the kidding and post-partum period, the NEFA concentration declined in higher BBR-treated groups (BBR2 and BBR4). Similarly, BHBA concentration at the kidding and thereafter is suppressed in BBR2- and BBR4-supplemented goats, which is more pronounced in BBR2. This suggests that CON does have a slightly larger energy deficiency. Likewise, the reduction in plasma levels of NEFA and BHBA in both BBR2 and BBR4 demonstrates an alleviated NEB, as it already known that plasma concentrations of BHBA and NEFA have been considered as effective indicators of energy status in transition goats (40) as well as cows (1).

The lower NEFA concentration in BBR2- and BBR4-fed goats may relate to BBR effects on improvement of energy status and reduction of body reserve usage. Likely, our previous finding (39) suggests that BBR may suppress lipolysis through enhancing feed intake and the endocrine responses that follow in periparturient dairy goats. In support of our findings, Shi et al. (41) showed that BBR can improve NEFA-induced lipid accumulation in a dose-dependent manner in bovine hepatocytes. Furthermore, data from rats demonstrated that BBR could protect high-fat diet-induced mitochondrial dysfunction (42). Teodoro et al. (43) reported that BBR could revert hepatic mitochondrial dysfunction in high-fat diet rats. These studies indicated that BBR could improve energy homeostasis and mitochondrial dysfunction in rodents. Additionally, the results of studies on type 2 diabetic patients further corroborate the favorable effects of BBR on glucose and insulin metabolism (15). The findings of Zhang and Chen (20) confirmed that BBR activates AMPK. It is well known that AMPK is a key energy-sensing system and acts as a regulator for body energy homeostasis (44).

The sudden rise in glucose concentration at the kidding in all groups is a normal response to parturition-induced endocrine changes that stimulate gluconeogenesis and lipolysis (45); however, this was not observed with BBR supplementation. The increments in plasma insulin concentration with BBR supplementation at the kidding were sustained until post-partum. The potential of BBR as a metabolic regulator agent has recently been shown both *in vitro* and *in vivo* (46). Zhou et al. (47) reported that treating rats with 50, 100, and 200 mg/kg BBR leads to increases in insulin expression, pancreatic B-cell regeneration, and plasma insulin. In the current investigation, the metabolic condition of both BBR2- and BBR4-supplemented doses suggests that BBR up to 50 mg/kg can effectively enhance insulin levels and diminish NEB and its plasma biomarkers in transition dairy goats.

### Biomarkers of Oxidative Stress and Antioxidant Status

Several recent studies provided increasing evidence that oxidative stress has been associated with several health and metabolic problems, especially during the transition period (7, 9). Our results demonstrated that supplementation with BBR (clearly BBR2 and BBR4) mitigated oxidative stress and had positive effects on antioxidant status, as indicated by enhanced TAC as well as SOD, GSH-Px, and CAT activity and depressed MDA level in almost all per- and post-partum periods in dairy goats. In support of earlier findings, Ghavipanje et al. (48) reported that goats fed *Berberis vulgaris* leaf (a natural rich source of BBR) had greater antioxidant capacity. However, there is a lack of direct study on the antioxidative effect of BBR in dairy animals, but experimental lines of evidence support the antioxidant activity of BBR, which reduces oxidative stress markers (12). Moghaddam et al. (19) reported that administration of 50 and 100 mg/kg BBR (doses corresponding to our study), significantly reduced MDA in rats. Similarly, a recent investigation showed that BBR addition to aflatoxin B1 and ochratoxin A contaminated broilers diet, partially restored levels of MDA and GSH-Px activity (18).

It has been shown *in vivo* that BBR administration upregulates enzymatic antioxidants as well. Zhou et al. (47) reported that daily oral ingestion of BBR restored the activity of SOD, catalase, and GSH-Px in the colon of rats exposed to azoxymethane. Another study showed that oral administration of BBR increased the activity of SOD in diabetic rats (16). Similarly, Tang et al. (49) showed that 100 and 200 mg/kg BBR for 21 days conserved SOD and GSH-Px activity and decreased MDA in Wistar rats. Moreover, it has been reported that mRNA expression level of SOD could be upregulated by BBR in diabetic mice, which played a role in antioxidative activity of BBR (30). Wang et al. (50) reported that BBR could inhibit oxidative stress *via* increased expression of uncoupling protein 2 (UCP2) in mice arteries.

Furthermore, it has been confirmed that BBR (50 μM to 1 mM) quenches free radicals in a concentration-dependent manner, as shown by its ability to scavenge radicals of 2,2-diphenyl-1- picrylhydrazyl (DPPH), nitric oxide (NO), 2,20-azino-bis (3-ethylbenzothiazoline-6-sulfonic acid) (ABTS), nitric oxide, and superoxide (13, 23). The molecular mechanisms of BBR in mitigation of oxidative stress seem to be related to multiple cellular pathways that were almost reviewed by Li et al. (23). The overall increase in enzymatic antioxidants from the pre- to post-partum period with BBR supplementation, in particular BBR2 and BBR4, may suggest that BBR elevates the antioxidant status of transition goats, which is vital for a successful shift from late pregnancy over the course of lactation.

### Biomarkers of Inflammation and Liver Functionality

Inflammatory conditions are signaled in the peripartum period often without clinical symptoms; hence, understanding of this phenomenon is important in order to improve the health and consequence performance of goats (4) as well as cows (51). The acute phase response of the liver to inflammation is accompanied by an increase in positive APP, along with a decrease in plasma negative APP, which is abundantly synthesized in the liver under normal condition (52). In our study, haptoglobin and ceruloplasmin among the positive APP were measured, which peaked at the kidding time. Overall, supplementation of both BBR2 and BBR4 at pre- and post-partum mitigates positive APP and enhances negative APP. Moreover, BBR increased plasma HDL concentration, as already well documented that apolipoprotein A, the basic protein for the synthesis of HDL, is considered a negative APP, and thus, the reduction in HDL could be caused by an inflammatory condition (53). Consistent with these results, Wang et al. (21) showed that supplementation of 100 mg/kg BBR suppressed pro-inflammatory cytokines in hepatitis-induced mice. Lou et al. (54) reported that BBR effectively inhibits IL-6 and TNF-α production in a concentration-dependent manner, which demonstrates its anti-inflammatory activity in hepatocytes. Hesari et al. (55) showed that BBR could prevent inflammation *via* reducing IL-1, TNFα, IL-6, and MCP-1, inhibiting PGE2 and COX-2 transcriptional activity in colon and other human cancer cells. BBR could reduce pro-inflammatory cytokines, acute phase protein, and infiltration of inflammatory cells in diabetic animal models (23). Additionally, the anti-inflammatory activity of BBR has been observed in different tissues like the serum, liver, adipose tissue, and kidney (15). Previous studies (22, 23) showed that the anti-inflammatory effects of BBR are also associated with its inhibitory effect on the mitogen-activated protein kinase (MAPK) signaling pathways, which were activated by inflammatory stimuli (23). Li et al. (23) demonstrated that BBR could reduce oxidative stress and inflammation through common cellular signaling pathways. Oxidative stress could stimulate the production of pro-inflammatory cytokines. On the other hand, pro-inflammatory cytokines could also increase the amount of ROS in cells and promote oxidative stress (56).

Inflammation could cause a decrease in PON and HDL synthesis in the liver, as well as an increase in bilirubin concentration (52). PON is associated with HDL and can inhibit the oxidative modification of low-density lipoprotein (LDL) (53), implying that PON may prevent inflammation in goats (6) as well as cows (51). The current finding indicated that the per- and post-partum BBR2 and BBR4 supplementation may elevate PON and HDL and mitigate bilirubin in goats during the transition period. In agreement, Cheng et al. (57) revealed that BBR stimulates PON1 transcription in human hepatoma cell lines *via* a JNK/c-Jun signaling pathway. Likewise, Malekinezhad et al. (18) provided lines of evidence on the anti-inflammatory activity of BBR in broilers. Because inflammation can be a cause of NEB as well as other confounding factors in the periparturition period (50), our results suggest that BBR may reduce inflammation in transition dairy goats.

## Conclusion

Overall, dietary supplementation with BBR reduced the plasma concentrations of NEFA and BHBA, which implies alleviated NEB. Plasma MDA levels were decreased at days −7, 14, and 21 relative to the kidding, whereas TAC levels were increased thereupon of the transition period following supplementation with 2 or 4 g/day BBR. Similarly, enzymatic antioxidants including TAC, SOD, GSH-Px, and CAT were enhanced by BBR2 and BBR4. Additionally, BBR supplementation (2 or 4 g/day BBR) appears to have benefited the immune system, due to decreased inflammatory biomarkers including ceruloplasmin and haptoglobin at kidding and thereafter. Taken together, this study showed the potential of BBR as a novel strategy to mitigate oxidative stress and inflammation in dairy goats during the transition period.

## Data Availability Statement

The raw data supporting the conclusions of this article will be made available by the authors, without undue reservation.

## Ethics Statement

The animal study was reviewed and approved by the Animal Welfare and Ethical Review Board of the Department of Animal Science, University of Birjand.

## Author Contributions

NG and MF: conceptualization and methodology. NG, MF, SF, and EV-B-P: validation. NG, SF, and SG: formal analysis. NG: investigation, writing—original draft preparation, and visualization. NG and SF: data curation. NG, MF, EV-B-P, and SG: writing—review and editing. MF: supervision. All authors have read and agreed to the published version of the manuscript.

## Funding

This study was supported by a grant from the Iran National Science Foundation (Grant Number: 97009560).

## Conflict of Interest

The authors declare that the research was conducted in the absence of any commercial or financial relationships that could be construed as a potential conflict of interest.

## Publisher's Note

All claims expressed in this article are solely those of the authors and do not necessarily represent those of their affiliated organizations, or those of the publisher, the editors and the reviewers. Any product that may be evaluated in this article, or claim that may be made by its manufacturer, is not guaranteed or endorsed by the publisher.
